# Pancreatitis-Associated Protein Does Not Predict Disease Relapse in Inflammatory Bowel Disease Patients

**DOI:** 10.1371/journal.pone.0084957

**Published:** 2014-01-09

**Authors:** Tiago Nunes, Maria Josefina Etchevers, Maria Jose Sandi, Susana Pinó Donnay, Teddy Grandjean, Maria Pellisé, Julián Panés, Elena Ricart, Juan Lucio Iovanna, Jean-Charles Dagorn, Mathias Chamaillard, Miquel Sans

**Affiliations:** 1 Department of Gastroenterology, Hospital Clinic of Barcelona (IDIBAPS/Centro de Investigació Bioméica en Red de Enfermedades Hepáicas y Digestivas [CIBEREHD]), Barcelona, Catalonia, Spain; 2 Centre de Recherche en Cancérologie de Marseille (CRCM), INSERM U1068, CNRS UMR 7258, Aix-Marseille Université and Institut Paoli-Calmettes, Parc Scientifique et Technologique de Luminy, Marseille, France; 3 University Lille Nord de France, F-59000, Lille, France; 4 Institut Pasteur de Lille, Center for Infection and Immunity of Lille, F-59019, Lille, France; 5 Centre National de la Recherche Scientifique, UMR8204, F-59021, Lille, France; 6 Institut National de la Santé et de la Recherche Médicale, U1019, Team 7, Equipe FRM, F-59019, Lille, France; 7 Department of Digestive Diseases, Centro Medico Teknon, Barcelona, Catalonia, Spain; Cincinnati Children's Hospital Medical Center, University of Cincinnati College of Medicine, United States of America

## Abstract

**Background:**

The pancreatitis-associated protein (PAP) is increased in the serum of active inflammatory bowel disease (IBD) patients and its levels seem to be correlated with disease activity. Our aim was to evaluate the usefulness of serum and fecal PAP measurements to predict relapse in patients with inactive IBD.

**Materials and Methods:**

We undertook a 12-month prospective study that included 66 Crohn's disease (CD) and 74 ulcerative colitis (UC) patients. At inclusion, patients were in clinical remission, defined by a Harvey-Bradshaw (HB) Index≤4 (CD) or a partial Mayo Score (MS)<3 (UC), along with a normal serum C reactive protein (CRP) and fecal calprotectin. Patients were followed every 3 months. Blood and stool samples were collected and a clinical evaluation was performed at each visit. Serum PAP and CRP levels as well as fecal concentrations of PAP and calprotectin were assessed.

**Results:**

Active CD patients had an increased mean serum PAP at the diagnosis of the flare (104.1 ng/ml) and 3 months prior to activity (22.68 ng/ml) compared with patients in remission (13.26 ng/ml), p<0.05. No significant change in serum PAP levels in UC and fecal PAP levels in CD and UC were detected during disease activity. In CD, serum PAP was a poor diagnostic predictor of disease activity, with an AUC of 0.69. In patients in remission, fecal PAP was barely detectable in UC compared with CD patients.

**Conclusion:**

Serum PAP is increased only in active CD patients, but this marker does not predict disease activity. Inactive UC patients have marked low levels of PAP in fecal samples compared with CD patients.

## Introduction

The pancreatitis-associated protein (PAP), also known as regenerating islet-derived protein 3 β (Reg3β) in mice, is a soluble calcium-dependent carbohydrate-binding protein which is expressed by intestinal epithelial cells.[1] It has been shown that colonization of germ-free mice with pathogens increases the expression PAP/Reg3β in the murine ileum and that this protein is directly bactericidal for gram-positive bacteria.[1] PAP/Reg3β also plays a protective role against intestinal translocation of gram-negative bacteria probably through interference with virulence mechanisms or host responses to these pathogens.[1] PAP/Reg3β triggers bacterial aggregation and displays bactericidal activity through its ability to directly bind some carbohydrate components of peptidoglycan.[2], [3] Recently, a protective role has been proposed for PAP/Reg3β in liver and pancreas, which is apparently independent of its bactericidal properties.[4], [5] *In vitro* studies showed that this protein plays an essential role in the negative regulation of cytokine signaling.[6], [7]

The gastrointestinal tract and the pancreas are major sources of PAP/Reg3β.[2], [8] In the colon, PAP/Reg3β is primarily secreted in the lumen by goblet cells and metaplastic Paneth cells[9] and in the *lamina propria* by intraepithelial lymphocytes[10]. Colonic expression of PAP/Reg3β is higher during inflammation-induced colorectal cancer and in the course of acute and chronic chemically-induced colitis in mice, whereas no significant changes are observed during the recovery phase.[11]–[13] In human subjects, PAP/Reg3β is over-expressed in the intestinal mucosa of patients with inflammatory bowel disease (IBD).[9], [11], [14] Importantly, clinical and endoscopic disease severity seems to correlate with serum PAP/Reg3β in parallel with C reactive protein (CRP) levels and erythrocyte sedimentation rate.[9]

The natural history of IBD is unpredictable and characterized by a succession of relapses and remissions. Furthermore, an overall 25–30% of therapy-refractoriness is seen, regardless of the available therapies.[15], [16] This usually results in a significant delay until the appropriate treatment is started and also in accumulation of adverse events. To help predicting clinical relapses in IBD, valuable non-invasive biomarkers are eagerly awaited. Previous preliminary data suggests that measurements of serum PAP/Reg3β have fine sensitivity and specificity in active ileal CD with positive and negative predictive values of 84% and 81%, respectively[14].

Collectively, our hypothesis is that an aberrant production of PAP/Reg3β takes place in IBD and that the measurement of PAP/Reg3β in humans could function as an inflammation marker with future clinical application. Our aim was to determine the clinical value of PAP/Reg3β testing in predicting clinical relapse in CD and UC patients.

## Materials and Methods

### Study design and definitions

This is a 12-month prospective study that included consecutive patients with inactive UC and CD recruited at the Hospital Clinic of Barcelona, in a 6-month period. All enrolled patients had an established diagnosis of IBD confirmed by standard radiological, endoscopic and histological features. Since endoscopy had not been performed before inclusion in most patients, the partial Mayo Clinic score (pMS) was used to establish remission in UC patients and the Harvey-Bradshaw (HB) Index was used for CD patients. Patients were considered in remission and suitable for study inclusion if they presented pMS<3 or HB≤4 and normal levels of CRP and calprotectin. Patients with previously known intestinal disorders or inflammatory diseases other than IBD, acute or chronic pancreatitis or chronic renal failure were excluded to avoid possible confounders in the PAP/Reg3β measurement.

During the follow-up, disease activity was evaluated by the same clinical scores used to define remission at inclusion (pMS or the HB index). At each visit, serum and fecal samples were collected to determine CRP and fecal calprotectin levels as standard biomarkers currently used in clinical practice. As the samples for CRP and calprotectin measurements were collected on the same day of the visit, they were not used to determine disease activity. For the PAP/Reg3ß measurements in sera and stool, blood and fecal samples were also collected at each visit and stored at −80 for posterior analysis.

After inclusion, patients were followed every 3 months until clinical disease activity was detected or the scheduled 12-month follow-up was completed. Data regarding demographics and disease description were collected from patient's charts. UC and CD characteristics were defined according to the Montreal Classification.

### PAP, Calprotectin and CRP measurements

Serum and fecal PAP/Reg3β was measured using a commercially available ELISA kit (Dynabio SA, Marseille, France) at the Institut Pasteur de Lille and results were expressed as ng of PAP/Reg3β per ml of serum or ng of PAP/Reg3β per mg of fecal material. The quantitative measurement of fecal calprotectin was performed at the Institut Pasteur de Lille by ELISA and results are given as ng/mg of stool sample. The CRP measurement was performed at the Hospital Clinic of Barcelona, and an elevated CRP was defined as higher than 0.8 mg/dL according to the hospitaĺs standard normal range. The measurement of all biological samples was investigator-blinded.

### Ethical issues

This Study was approved by the Ethics Committee of Hospital Clinic of Barcelona, Spain. All patients gave their written informed consent before enrolment. Data was anonymously analysed to preserve patient's confidentiality.

### Statistical analysis

Qualitative variables were expressed using frequencies. Continuous variables were expressed using median and interquartile range for demographics and mean with standard error of the mean (SEM) for laboratory measurements. A Kolmogorov–Smirnov test was used to evaluate whether serum and fecal PAP/Reg3ß, CRP and calprotectin values followed a normal distribution. Student's t tests or the Wilcoxon–Mann–Whitney test were performed for these continuous variables. A P-value<0.05 was considered statistically significant. The global yield of the serum PAP/Reg3ß to predict IBD relapse was calculated using the area under the ROC (receiver operating characteristic) curve.

## Results

### Patientś characteristics, follow-up and relapse rate

UC patients. 74 patients were enrolled. About 35% of the recruited UC patients had disease limited to the rectum, 42% extensive disease and 23% left-sided involvement. Additional data on patientś characteristics are shown in **Table 1**. 67 patients completed the scheduled 12-month follow-up. 7 patients were excluded: 5 patients as a result of initiating treatment for disease activity on their own before stool and serum sample collection, 1 refused to continue in the study and 1 moved to a different city.

**Table 1:** Main characteristics of CD and UC patients.

Characteristics	CD patients	UC patients
**Number of patients**	66	74
**Male**	35 (53)	35 (47)
**Age at diagnosis (years)**	30 (25–44)	34 (27–45)
**Disease duration (months)**	90 (53–120)	90 (48–149)
**Disease location**		
Ileal/Proctitis	34 (52)	26 (35)
Colonic/Left-sided	10 (15)	17 (23)
Ileocolonic/Extensive	21 (32)	31 (42)
Upper GI	1 (1)	
**Perianal disease**	10 (15)	
**Extraintestinal manifestations**	17 (26)	19 (26)
**Disease behavior**		
Inflammatory	36 (55)	
Stricturing	19 (29)	
Penetrating	11 (17)	
**Bowel resection**	20 (30)	
**Maintenance therapy**		
5-ASA	1 (2)	47 (39)
Immunosuppressants	32 (48)	20 (15)
Anti-TNF	6 (9)	2 (2)
No treatment	29 (44)	7 (6)

Categorical variables are represented as frequencies (percentages) and continuous variables are represented as median and inter-quartile range.

CD patients. 66 patients were enrolled in the longitudinal study and 62 completed the follow-up. 34 had ileal disease (52%), 10 colonic (15%), 21 Ileocolonic (32%) and 1 patient upper GI disease (1%). Regarding disease behavior, most patients had non-stricturing non-penetrating disease. Additional information on patientś characteristics are also shown in **Table 1**. In addition to the 62 recruited patients who continued in the study, 4 patients were excluded: 1 as a result of not reporting a flare and auto-medication and 3 refused to continue in the study.

**Figure 1:**
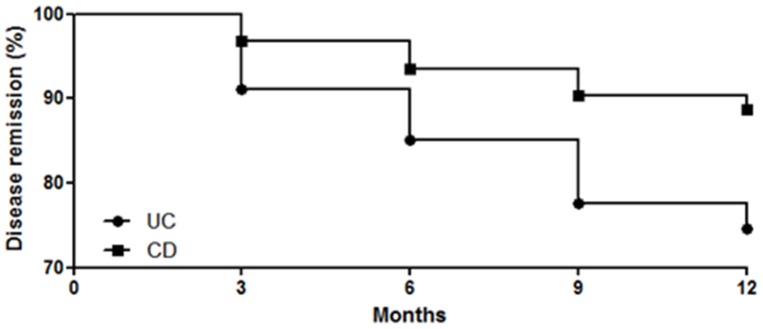
Remission rates in CD (n = 62) and UC (n = 67) patients over the 12-month period of follow-up. 17 out of the 67 UC patients (25%) who completed the follow-up had disease activity with a mean pMS score of 8. In the CD leg, 7 out of the 62 patients (11%) had clinical disease activity with a mean HB index of 6.

Relapse rates. Overall, 7 CD (11.2%) and 17 UC (25.3%) patients who completed the follow-up had disease activity during the 1-year follow-up period, **Figure 1**. Most patients with disease activity in the CD group had mild disease according to the HB index (5−7 points) and only 2 patients had moderate disease (8−16 points) with no patients with severe flare (>16 points). For UC patients, all patients had a moderate-severe flare with pMS always higher than 5 points.

### Mean serum and fecal PAP/Reg3β, CRP and calprotectin in UC

**Figure 2:**
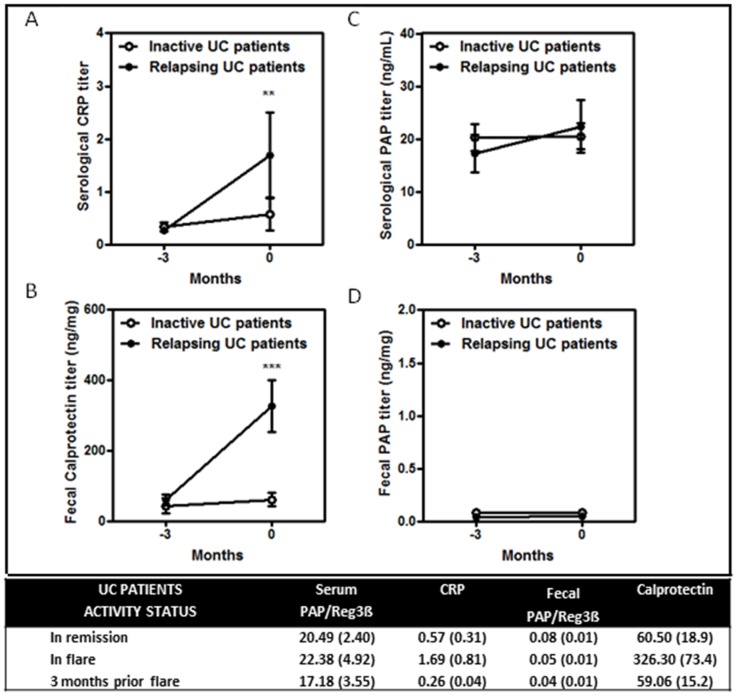
Graphics for mean serum and fecal PAP/Reg3β, CRP and calprotectin levels in UC patients at disease relapse and 3 months before the diagnosis of disease activity. In the table, mean values and standard error of the mean of serum and fecal PAP/Reg3β (ng/ml in serum and ng/mg in stool), CRP (mg/dL) and calprotectin (ng/mg) in UC patients in remission, flare and 3 months before the episode of disease activity.

Mean values for serum and fecal PAP/Reg3β, CRP and calprotectin in UC are shown in **Figure 2**. Mean serum (**Figure 2C**) and fecal (**Figure 2D**) PAP/Reg3β levels were similar in relapsing and inactive UC patients. In contrast, serum CRP and fecal calprotectin levels (**Figures 2A and 2B**) were higher at disease relapse compared with patients in remission (p<0.005). In addition, no differences were observed in serum and fecal PAP/Reg3β between active and relapsing UC patients at any time point, including the visit prior to flare diagnosis.

### Mean serum and fecal PAP/Reg3β, CRP and calprotectin in CD

**Figure 3:**
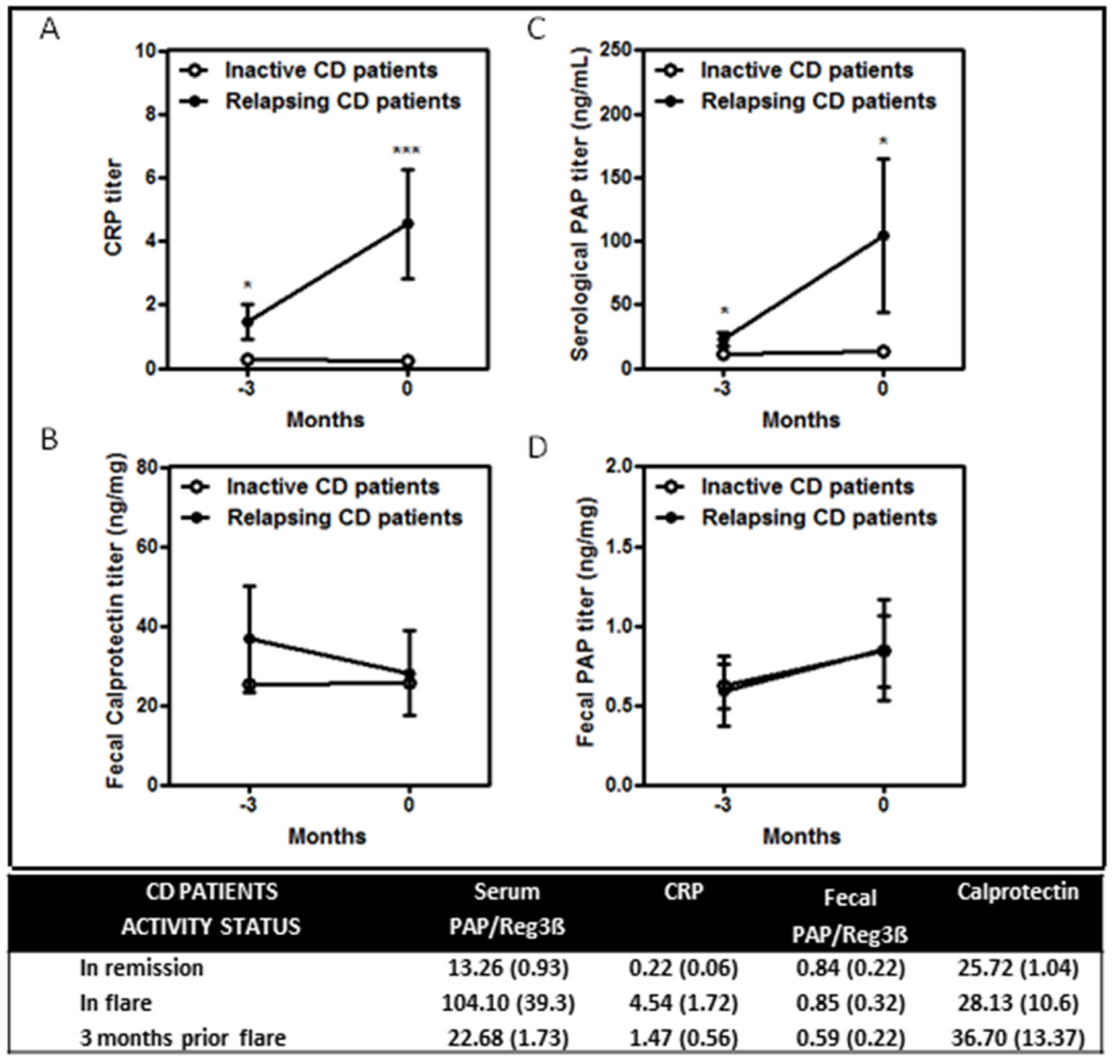
Graphics for mean serum and fecal PAP/Reg3β, CRP and calprotectin levels in CD patients at disease relapse and 3 months before the diagnosis of disease activity. In the table, mean values and standard error of the mean of serum and fecal PAP/Reg3β (ng/ml in serum and ng/mg in stool), CRP (mg/dL) and calprotectin (ng/mg) in CD patients in remission, flare and 3 months before the episode of disease activity.

Mean values for serum and fecal PAP/Reg3β, CRP and calprotectin in CD are shown in **Figure 3**. Serum CRP (**Figure 3A**) and PAP/Reg3β (**Figure 3C**) levels were higher at disease relapse compared with patients in remission (p<0.0005 and p<0.05 respectively). Likewise, serum CRP (**Figure 3A**) and PAP/Reg3β (**Figure 3C**) were also increased at the visit prior to disease relapse (p<0.05). In contrast, fecal calprotectin (**Figure 3B**) and fecal PAP/Reg3β (**Figure 3D**) levels were similar in relapsing and inactive CD patients.

### Receiver operating characteristics (ROC) analysis

**Figure 4:**
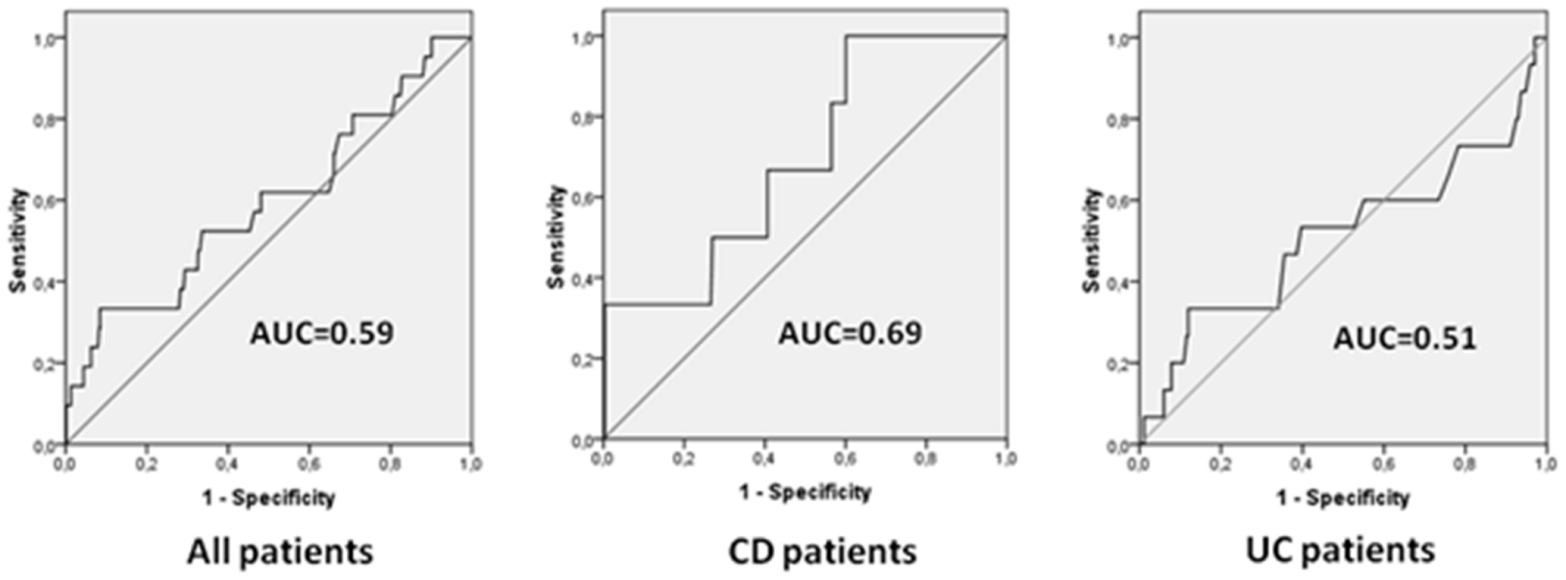
Area under the receiver operating characteristic (ROC) curve to predict inflammatory bowel disease relapse using serum PAP/Reg3β determination in CD, UC and in the combined group of IBD subjects.

Given the differences regarding serum PAP/Reg3β levels between CD patients in flare and remission, the efficacy of serum PAP/Reg3β as a diagnostic marker for intestinal inflammation was evaluated. The area under the ROC curve to predict CD relapse using PAP/Reg3β was 0.69 (**Figure 4**). The best global cutoff point was 16.85 ng/ml (sensitivity 50%, specificity 73%). ROC curves for serum PAP/Reg3β in UC patients and in IBD patients in general are also shown (**Figure 4**).

### Mean serum and fecal PAP/Reg3β during follow-up in patients in remission

**Figure 5:**
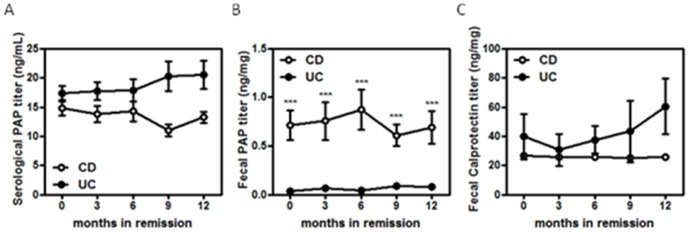
Graphics representing the mean serum and fecal PAP/Reg3β and calprotectin levels at different time points over time in UC and CD patients in remission.

During remission, serum PAP/Reg3β (**Figure 5A**), fecal PAP/Reg3β (**Figure 5B**) and calprotectin levels were stable over time. Surprisingly, fecal PAP/Reg3β (**Figure 5B**), but not systemic (**Figure 5A**), was barely detectable in inactive UC patients when compared with CD patients. In contrast, no differences in fecal calprotectin levels were observed between UC and CD patients during remission (**Figure 5C**).

## Discussion

Growing evidence indicates that mucosal healing is a surrogate marker of sustained remission in CD and UC and should be the ultimate therapeutic goal in the management of IBD patients.[17] Endoscopic evaluation is still the gold standard for assessment of mucosal healing though the procedure is invasive and costly.[18] As alternative non-invasive inflammatory markers, increased fecal levels of calprotectin and lactoferrin indicate intestinal inflammation of any cause, showing good positive predictive values for endoscopically active disease in IBD.[18] As for serum CRP, however, these fecal parameters lack specificity for IBD-related mucosal inflammation, being also elevated in the infectious involvement of the gut.[19]–[21] In addition, many patients in clinical practice have endoscopically active inflammation and fecal protein and CRP levels within the normal range.[18] New and better non-invasive diagnostic tools, therefore, are urgently needed.

The notion that intestinal PAP/Reg3β production is increased in active IBD patients had already been suggested by studies measuring PAP/Reg3β mRNA in intestinal tissue.[11], [22], [23] In addition, PAP/Reg3β levels were previously shown to be elevated in sera of patients with IBD compared with controls.[9], [14] In the most recent study, performed by Gironella *et al*, UC and CD patients had higher serum PAP/Reg3β levels than controls even in disease remission. In contrast to current non-invasive diagnostic markers available in clinical practice, the authors showed that the increase in serum PAP/Reg3β followed in parallel with disease activity and it seemed to be specific for IBD as protein levels were not elevated in subjects with infectious diarrhea.[9] These previous findings led us to hypothesize that higher levels of PAP/Reg3β could function as a disease activity serological marker specific for IBD-related inflammation and that prospective measurements of this protein could predict future disease activity in patients in clinical remission.

In keeping with these previous data, serum PAP/Reg3β levels were higher at disease relapse than in inactive CD patients. Importantly, patients with active disease also had increased levels of PAP/Reg3β compared with subjects in remission at the visit prior to disease flare suggesting that this marker could help predict future clinical relapse. As serum PAP/Reg3ß, serum CRP was also elevated at these two time points, at clinical recognizable disease flare and 3 months before activity was diagnosed. Nevertheless, in the Receiver operating characteristics (ROC) analysis, serum PAP/Reg3ß had poor predictive diagnostic accuracy to detect disease activity in patients with CD. We could not confirm, therefore, the findings of a previous study performed by Desjeux *et al*. which found that a serum PAP concentration above 50 ng/mL indicated active CD with a good accuracy (sensitivity of 60% and specificity of 94%).[14] In this regard, the small number of CD patients relapsing during the 1-year follow-up in our cohort might be accountable for these differences.

In contrast to CD, an increase in PAP/Reg3β levels was not detected in sera of active UC subjects compared with patients in remission. Contrary to the study by Gironella *et al* [9] which found higher levels of serum PAP/Reg3β in random UC samples compared with controls, the present study has the strength of being the first to follow patients prospectively with several PAP/Reg3β measurements during different time points. One can hypothesize that, even though an elevated clinical score was measured, patients with UC in this series did not have actual intestinal inflammation. In our study, however, disease activity in UC patients was not only documented with elevation in the clinical activity score but this group also had significant elevations of serum CRP and fecal calprotectin compared with patients in remission. Our results support the notion that there is no role for the measurement of PAP/Reg3β in sera of UC patients. In keeping with this finding, Desjeux *et al*. found that an increase in serum PAP/Reg3β was specific for ileal inflammation as CD patients with active colonic disease had normal levels of PAP/Reg3β compared with controls.[14]

When it comes to the utility of PAP/Reg3β measurements in stool, results are more clearly negative. In this regard, there were no differences in fecal PAP/Reg3β levels in CD regardless of disease activity. Surprisingly, though active CD patients had a marked increase in CRP compared with subjects in remission, no differences with respect to fecal calprotectin were found at any time point. The lower sensitivity of calprotectin to assess proximal gut inflammation [18] (most active patients presented ileal or upper GI inflammation) and the low number of CD patients who had clinical activity in our cohort could account for these results. The fact that there were also no differences in fecal PAP/Reg3β levels between active UC patients and subjects in remission further indicates that there is no role for fecal PAP/Reg3β in the assessment of IBD-related gut inflammation.

Overall, we do not recommend PAP/Reg3β testing in patients with UC. In contrast, in CD patients, the low relapse rate in our cohort makes it difficult to definitely exclude the potential utility of PAP/Reg3β, especially for serological measurements. Given that patients were in remission at inclusion, it is likely that CD patients with mild disease course were selected. Accordingly, most active CD patients had only mild disease activity and no patient with a severe flare was detected.

Our study is the first to report on the patterns of fecal and serum PAP/Reg3β levels over time in IBD in remission. In these patients, serum and fecal PAP/Reg3β levels were very stable during the 12-month follow-up. Interestingly, a constant decreased intraluminal secretion of PAP/Reg3β in UC patients with no disease activity was observed. As mentioned before, this protein is believed to have an important role in the homeostasis of the intestine. It has been suggested that an increased production of PAP/Reg3β probably related to hyperplasia/metaplasia of Paneth cells might have anti-inflammatory effects in chronic intestinal inflammation with a potential protective role in the pathogenesis of IBD.[9]

Two mechanisms have been proposed to explain these anti-inflammatory properties, both through down-regulation of NFkB. First, NFkB-dependent secretion of pro-inflammatory cytokines in the intestine of IBD subjects was shown to be down-regulated by PAP/Reg3β in endothelial cells, epithelial cells and monocytes.[9] Second, it has been demonstrated that PAP/Reg3β can inhibit leukocyte recruitment into the bowel by monitoring up-regulation of E-selectin, ICAM-1, and VCAM-1.[9] Sustained low intraluminal levels of PAP/Reg3β, therefore, could impact the immunological balance of the intestinal mucosa in these patients, contributing to the pathogenesis of bowel chronic inflammation. These interesting findings linking for the first time UC and low intraluminal secretion of PAP/Reg3β require further evaluation.

In conclusion, our study further confirms that serum PAP/Reg3β levels are increased in active CD patients and show that these serum levels are also elevated 3 months prior to clinical activity. Even though no significant predictive value was determined for serum PAP/Reg3β in CD, further prospective studies including endoscopic evaluation, with larger number of patients are needed to definitely exclude this marker as a possible diagnostic tool in this setting. On the contrary, fecal PAP/Reg3β concentrations are not associated with disease activity in both CD and UC patients and have no clinical utility. Constant marked decreased levels of PAP/Reg3β in fecal samples of UC patients in remission were detected requiring further work on the role of this alleged anti-inflammatory protein in the pathogenesis of IBD.
